# Coexistence of Flexibility and Stability of Proteins: An Equation of State

**DOI:** 10.1371/journal.pone.0007296

**Published:** 2009-10-09

**Authors:** Marina de Leeuw, Shlomi Reuveni, Joseph Klafter, Rony Granek

**Affiliations:** 1 Department of Biotechnology Engineering, Ben-Gurion University of The Negev, Beer Sheva, Israel; 2 School of Chemistry, Tel-Aviv University, Tel-Aviv, Israel; 3 Freiburg Institute for Advanced Studies (FRIAS), Albert-Ludwig-Universität Freiburg, Freiburg, Germany; Fox Chase Cancer Center, United States of America

## Abstract

We consider a recently suggested “equation of state” for natively folded proteins, and verify its validity for a set of about 5800 proteins. The equation is based on a fractal viewpoint of proteins, on a generalization of the Landau-Peierls instability, and on a marginal stability criterion. The latter allows for coexistence of stability and flexibility of proteins, which is required for their proper function. The equation of state relates the protein fractal dimension 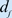, its spectral dimension 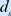, and the number of amino acids *N*. Using structural data from the protein data bank (PDB) and the Gaussian network model (GNM), we compute 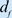
and 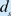 for the entire set and demonstrate that the equation of state is well obeyed. Addressing the fractal properties and making use of the equation of state may help to engineer biologically inspired catalysts.

## Introduction

Proteins are one of the major components of living cells. They constitute more than half of the cell's dry weight, and are responsible for the execution of most cellular functions required for life, including among others, catalysis and molecular recognition within and between cells and their surroundings. Understanding the relationships between structure, internal dynamics, and enzymatic activity at the single-molecule level could pave new ways to manipulate individual molecules.

Two seemingly conflicting properties of native proteins, such as enzymes and antibodies, are known to coexist. While proteins need to keep their specific native fold structure thermally stable, the native fold displays the ability to perform large amplitude conformational changes that allow proper function [1]. This conflict cannot be bridged by compact objects which are characterized by small amplitude vibrations [2]. Recently, however, it became evident that proteins can be described as fractals; namely, geometrical objects that possess self similarity [3], [4]. Adopting the fractal point of view to proteins makes it possible to describe within the same framework essential information regarding topology and dynamics.

Based on the fractal viewpoint, we have recently derived a universal equation of state for protein topology. The same fractal viewpoint allows describing the near equilibrium dynamics of native proteins. We have recently shown that it leads to anomalous dynamics [5]. For example, the autocorrelation function of the distance between two 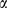-carbons on a protein is predicted to decay anomalously, first, at short times, as 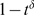 and later, at long times, as 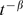, where 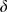 and 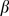 are exponents that depend on various fractal dimensions. This type of relaxation has been recently observed in single molecule experiments [6], [7]. Closely related is the anomalous diffusion of an 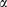-carbon that is predicted by the fractal model, where the mean square displacement is found to increase as 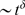. Such a behavior has also been recently observed in molecular dynamics simulations [8].

Natively folded proteins can be characterized by broken dimensions: the fractal and spectral dimensions [2], [4], [5], [9]–[12]. The mass fractal dimension 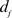 describes the spatial distribution of the mass within the protein via the scaling relation 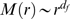, where 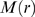 is the mass enclosed in a sphere of radius 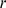 [3]. The spectral dimension 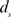 governs the density of low frequency vibrational normal modes *via* the scaling relation 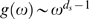, where 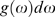 is the number of modes in the frequency range 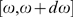 [13]. While for regular three dimensional (3D) lattices both 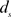 and 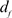 coincide with the usual dimension of 3, for proteins it is usually found that 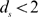 and 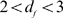, leading to an excess of low frequency modes and a more sparse fill of space [2], [4], [12]. Importantly, the regime 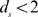 is associated with the so-called Landau-Peierls instability, where the amplitude of vibrations increases with the number of residues *N* [14], [15]. As this amplitude overcomes a threshold value, it may cause the protein to unfold [2], [12].

The Landau-Peirels instability is most readily derived using the density of states. The static mean square displacement (MSD) of an 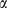-carbon, which is essentially the so-called *B_i_*-factor, averaged over all 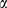-carbons of the protein, may be expressed as
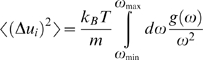
(1)where *m* is the average mass of an amino acid. Since 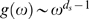, it follows that if 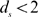 the integral diverges with the lower bound 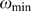. The latter depends on the protein radius of gyration 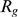 and the number of residues 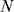 as 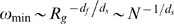. This leads to 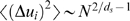, which increases with 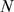 for 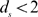. In particular, the static MSD of *surface* residues has been argued to grow as
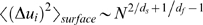
(2)


We have proposed a marginal stability criterion [16], in which most proteins “exploit” the Landau-Peierls instability to attain large amplitude vibrations, which is required for their proper function, yet maintaining their native fold. Thus proteins are assumed to exist in a thermodynamic state close to the edge of unfolding. Based on this and the Landau-Peirels instability of the surface residues, Eq. (2), a general equation of state has been proposed that relates between the spectral dimension 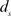, the fractal dimension 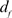, and the number of amino acids along the protein backbone *N*:
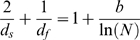
(3)where *b* is a molecular fit parameter depending on the temperature *T*, the GNM spring constant γ, and the GNM cutoff *R_c_*: b≈ln(γ*R_c_* ^2^/*k_B_T*)) [12]. It has been shown that this equation is obeyed by about 500 proteins regardless of their source or function [12]. In the present study we check the validity of Eq. (3) for a much larger set of over 5,000 proteins, using a range of statistical methods, and show that also for this very large set Eq. (3) is beautifully fulfilled. This supports the marginal stability criterion that led to this equation.

## Methods

We have used all data files present in the Protein Data Bank (PDB) [17] and filtered out proteins exceeding 95% sequence identity and proteins that have ligands, RNA, or DNA. We have also removed incomplete data files, files that contained data of the 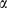-carbons alone, and also files of proteins smaller than 100 amino acids that are too small to be characterized as fractals. With this screening the set has been reduced to 5793 proteins.

**Figure 1:**
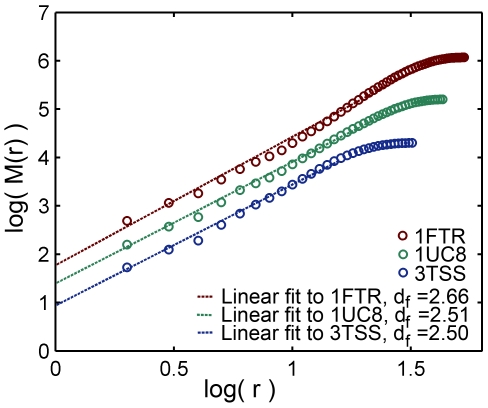
Fractal dimension. The fractal dimension of three selected proteins: 1FTR (1184 amino acids, 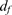 = 2.66), 1UC8 (505 amino acids, 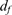 = 2.51) and 3TSS (190 amino acids, 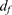 = 2.50). The mass 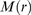 enclosed in concentric spheres of radius 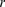 is plotted against 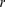 (measured in units of Å) on a log-log scale and the slope determines the fractal dimension, 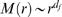. The plots of 1FTR and 1UC8 were shifted along the y axis (+1 and +0.5 respectively) for clarity.

The fractal and spectral dimensions were calculated for all 5793 proteins in similar ways to the procedure described by [12]. Finding the protein center of mass and placing the origin of coordinates at the ten nearest 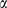-carbons, the mass was calculated as a function of the distance *r* on a log-log scale. The fractal dimension 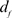 has been obtained as the slope of this plot for distances below the protein gyration radius *R_g_*, averaged over the ten origin of coordinates, see examples in Fig. 1. It should be noted that when a few alternative locations of an atom are given, only the “*A*” location (usually the most abundant one) has been used.

**Figure 2:**
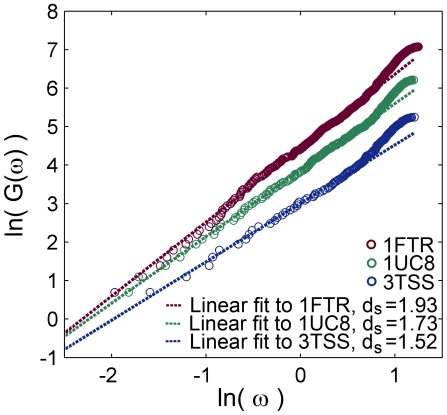
Spectral dimension. The spectral dimension of three selected proteins (same proteins as in Fig. 1): 1FTR (1184 amino acids, 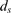 = 1.93), 1UC8 (505 amino acids, 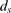 = 1.73) and 3TSS (190 amino acids, 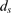 = 1.52). The cumulative density of normal modes 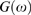 is plotted against the frequency 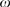 (measured in units of the spring natural frequency) on a log-log scale and the slope determines the spectral dimension, 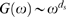.

To find the spectral dimension 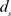, we calculate the cumulative density of normal vibrational modes 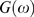, 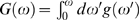, representing the number of modes up to a frequency 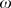. To obtain the vibrational modes, we used a frequently applied elastic model for protein vibrations, the Gaussian network model (GNM) [12], [18]–[23]. Two values were taken for the interaction distance cutoff *R_c_*, that describes the range of the interaction between an 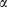-carbons pair, *R_c_* = 6 Å and *R_c_* = 7 Å. Plotting on a log-log scale 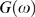 against the frequency 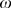, the slope in the low frequency range (containing about 24% of the modes, independent of the protein type or size *N*) defines 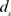, i.e. 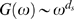, see examples in Fig. 2 for the case *R_c_* = 6 Å.

To deal with the large number of proteins in this set, both procedures were automated using suitable computer codes. The automatically calculated spectral dimension values were compared (for the case *R_c_* = 7 Å) to the manually obtained values for the set of 543 studied in [12]. We found almost vanishing mean of the difference between the two results (0.0034), showing that the error is statistical, and a low standard deviation (0.083), suggesting good agreement between the two methods of calculation.

In order to generally check for correlations between 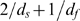 and 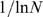, simple regression was conducted (using SPSS). This shows statistical significance with p<0.001 and very high *F*-test values (*F*(1,5791) = 3263 and *F*(1,4247) = 2120 for R_c_ = 6 Å, *F*(1,5791) = 4059 and *F*(1,3888) = 2314 for R_c_ = 7 Å).

## Results

**Figure 3:**
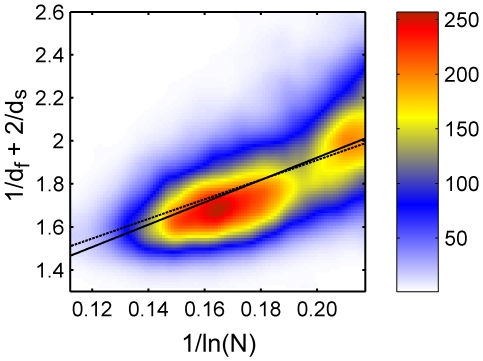
Full data set for 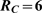 Å, colored histogram. The values of 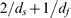 against 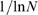 plotted for the full data set (5793 proteins) with 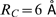 Å. The data is presented using a smoothed colored histogram based on a 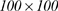 grid, see the color scale on the right (low density areas colored blue and high density red). The data was fitted to Eq. (3) (dashed line) and to Eq. (4) (full line).

**Figure 4 pone-0007296-g004:**
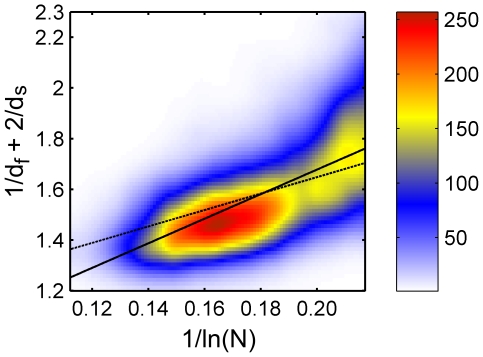
Full data set for 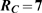 Å, colored histogram. Same as in Fig. 3 but for 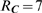 Å.

The results for the whole set appear in the supporting information S1 and are shown in Figs. 3 and 4 (for *R_c_* = 6 Å and 7 Å, respectively), where we plot the combination 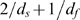 against 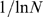. In order to present the whole set of data, we designed a (smoothed) colored histogram based on a 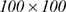 grid, where a pixel color represent the number of proteins associated with the pixel. The data is first fitted to Eq. (3) (dashed lines). This leads to b = 4.555 for *R_c_* = 6 Å (correlation coefficient cc = 0.596), see Fig. 3, and b = 3.242 for *R_c_* = 7 Å (cc = 0.605), see Fig. 4. Using b≈ln(γ*R_c_* ^2^/*k_B_T*), with *k_B_T*/γ in the range 0.5 Å^2^ to 2 Å^2^, we can estimate *b* to be in the range 3 to 5. The value of *b* is within the expected range.

We also fitted the data to an equation resembling Eq. (3) but in which the value “1” is replaced by a free parameter *a*:
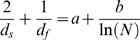
(4)


This is done in order to verify if the free fit recovers the value *a* = 1. The results of this fit are also shown in Figs. 3–4 (full lines), and yield a = 0.884 and b = 5.197 for *R_c_* = 6 Å (Fig. 3, cc = 0.600), and a = 0.710 and b = 4.841 for *R_c_* = 7 Å (Fig. 4, cc = 0.642). Remarkably, the colored histogram shows a ridge roughly centered at the best fitting theoretical lines.

**Figure 5 pone-0007296-g005:**
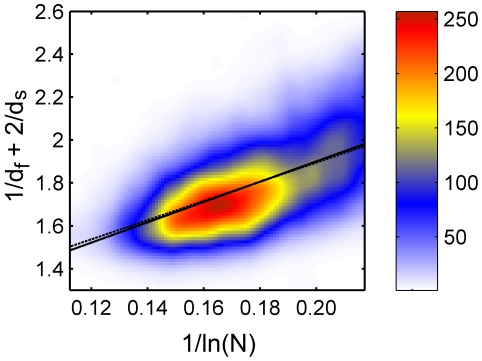
High precision data set for 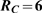 Å, colored histogram. The values of 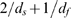
against 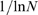 plotted for the refined subset of increased precision for 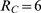 Å (4249 proteins), using a colored histogram (same as in Fig. 3).The data was fitted to Eq. (3) (dashed line) and to Eq. (4) (full line); the two lines are almost indistinguishable.

**Figure 6 pone-0007296-g006:**
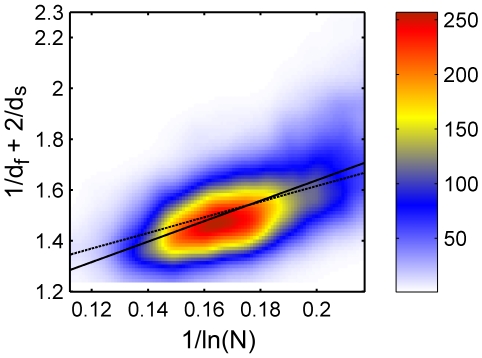
High precision data set for 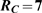 Å, colored histogram. Same as in Fig. 5 but for the refined subset of increased precision for 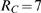 Å (3890 proteins).

To improve the accuracy of the analyses, a subset was constructed containing only those proteins whose both 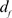 and 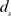 values have been determined with a *very high precision*, such that the squared correlation coefficients for the power-law fits of both 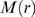 and 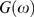 were in the range R^2^>0.99. Accordingly, this subset for *R_c_* = 6 Å (containing 4249 proteins) is not identical to the subset for *R_c_* = 7 Å (containing 3890 proteins), see the supporting information S1 for details. The results are presented in Figs. 5–6. Fitting to Eq. (3) (dashed lines) leads to b = 4.476 for *R_c_* = 6 Å (Fig. 5, cc = 0.576) and b = 3.078 for *R_c_* = 7 Å (Fig. 6, cc = 0.593). Fitting the data to Eq. (4) (full lines), yields a = 0.952 and b = 4.747 for *R_c_* = 6 Å (Fig. 5, cc = 0.577), and a = 0.833 and b = 4.031 for *R_c_* = 7 Å (Fig. 6, cc = 0.611).

Although the data analysis presented in Fig. 3–6 appears complete, it fails to give equal weight to proteins of different sizes. All four different data sets used above are very rich in proteins of small (100–200 residues) and intermediate size, a consequence of their abundance in nature, while being poor in large proteins. Yet, the linear regression presented in Figs. 3–6 gives each protein an equal weight. Thus, while the small/intermediate size proteins are spread over a relatively limited range of *N*, they are overwhelming the linear regression, which is undesirable.

**Figure 7 pone-0007296-g007:**
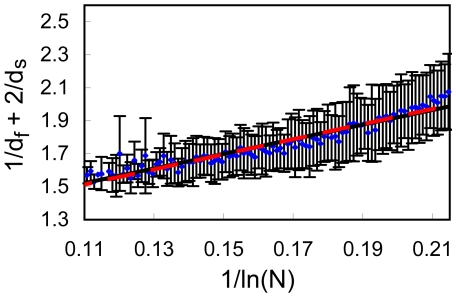
Full data set for 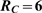 Å, division into bins. The values of 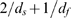 against 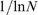 plotted for the full data set (5793 proteins) with 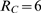 Å. The values of 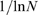 were divided into 100 equally sized bins. For each bin we show the average value of 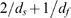 and the error bar presents its standard deviation. The data was fitted to Eq. (3)(dashed red line) and to Eq. (3) (full black line); the two lines are almost indistinguishable.

**Figure 8 pone-0007296-g008:**
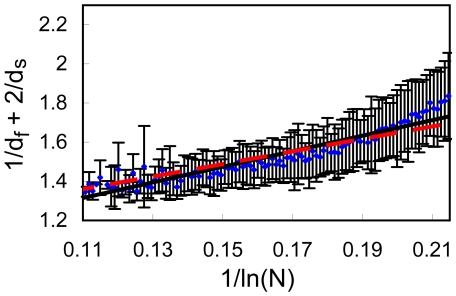
Full data set for 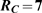
 Å, division into bins. Same as in Fig. 7 but for 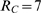 Å.

**Figure 9 pone-0007296-g009:**
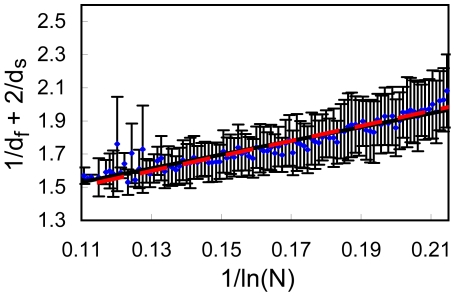
High precision data set for 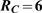 Å, division into bins. Same as Fig. 7 but for the refined subset of increased precision for 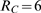 Å (4249 proteins).

**Figure 10:**
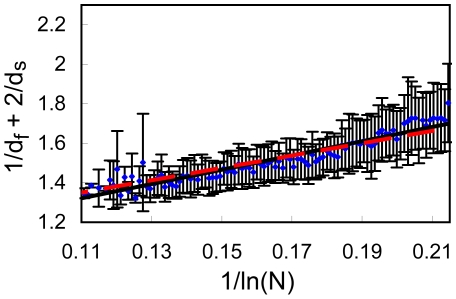
High precision data set for 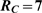 Å, division into bins. Same as Fig. 7 but for the refined subset of increased precision for 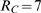 Å (3890 proteins).

To circumvent this artifact, we have separated the *x*-axis (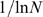) into 100 bins. For each bin we calculate the mean value of 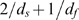. The error of 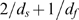 for each bin is estimated as the standard deviation of this value. The results are summarized in Figs. 7, 8, 9, 10.

Results from the full set of 5793 proteins are presented in Figs. 7–8. Fitting to Eq. (3) (dashed lines) leads to b = 4.580 for *R_c_* = 6 Å (Fig. 7, cc = 0.957) and b = 3.212 for *R_c_* = 7 Å (Fig. 8, cc = 0.928). Fitting the data to Eq. (4) (full lines), yields a = 1.026 and b = 4.429 for *R_c_* = 6 Å (Fig. 7, cc = 0.958), and a = 0.870 and b = 3.977 for *R_c_* = 7 Å (Fig. 8, cc = 0.946). Note that all lines pass through almost all error bars, a remarkable result.

In Figs. 9–10 we present results from the high precision subset of 4249 proteins. Fitting to Eq. (3) (dashed lines) leads to b = 4.535 for *R_c_* = 6 Å (Fig. 9, cc = 0.941) and b = 3.124 for *R_c_* = 7 Å (Fig. 10, cc = 0.937). Fitting the data to Eq. (4) (full lines), yields a = 1.065 and b = 4.155 for *R_c_* = 6 Å (Fig. 9, cc = 0.945), and a = 0.917 and b = 3.609 for *R_c_* = 7 Å (Fig. 10, cc = 0.946). Here, as well, all lines pass through almost error bars. This refined analysis gives an even stronger support to Eq. (3).

## Discussion

All correlation coefficients mentioned above (Figs. 3–10) are considered excellent. In addition, the values of *a* are close to the theoretically predicted value *a* = 1, similar to the set of 543 proteins studied by [12]. In particular, the fits of the data to Eq. (4) for all data sets belonging to *R_c_* = 6 Å (shown in Figs. 3,5,7 and 9) yields *a* values that are remarkably close to 1. The distribution of the data in all four sets appears as a ridge that is roughly centered at the best fitting theoretical lines (Figs. 3–6), and when the binning procedure is being used, all lines pass well through the error bars (Figs. 7–10). We believe that these results strongly confirm the universal behavior described by Eq. (3), thereby supporting the theoretical arguments leading to this equation.

Importantly, *a* is found to be particularly close to 1 when the binning procedure is introduced, in which we analyze the mean value of 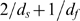, for a given *N*, for its dependence on *N*. In these cases we also obtain remarkably good correlation coefficients, significantly better than those obtained without binning. This suggests that, as a group, proteins follow the equation of state, although the error bars indicate that there are other factors present that cause deviations from the equation. These factors could be related to the protein specific structure and/or function.

The distribution of the data in all four sets appears as a ridge that is roughly centered at the best fitting theoretical lines (Figs. 3–6), and when the binning procedure is being used, all lines pass well through the error bars (Figs. 7–10). We believe that these results strongly confirm the universal behavior described by Eq. (3), thereby supporting the theoretical arguments leading to this equation.

To conclude, our analysis confirms the fractal nature of proteins and supports the predicted universal equation of state (3). This suggests that the majority of proteins in the PDB exist in a marginally stable thermodynamic state, namely a state that is close to the edge of unfolding. This could be related to the fact that enzymes require flexibility and large internal motion to function properly [1]. We suggest that Eq. (3) can be used as a tool in the design of artificial enzymes [24]. Interestingly, fractal-like properties have also been suggested to appear in the configuration space of peptides [25].

## Supporting Information

Supporting Information S1 A file containing the mass fractal dimension *d_f_* and spectral dimension *d_s_* of all proteins analyzed, divided into the four data sets described in the text: (i) GNM cutoff length 6 Å, (ii) GNM cutoff length 7 Å, (iii) GNM cutoff length 6 Å, a subset with high precision values of *d_f_* and *d_s_*, and (iv) GNM cutoff length 7 Å, a subset with high precision values of *d_f_* and *d_s_*.
(2.84 MB XLS)
